# VIBES: A consensus subtyping of the vaginal microbiota reveals novel classification criteria

**DOI:** 10.1016/j.csbj.2023.11.050

**Published:** 2023-11-30

**Authors:** Diego Fernández-Edreira, Jose Liñares-Blanco, Patricia V.-del-Río, Carlos Fernandez-Lozano

**Affiliations:** a Department of Computer Science and Information Technologies, Faculty of Computer Science, CITIC-Research Center of Information and Communication Technologies, Universidade da Coruña, A Coruña, Spain; b University of A Coruña, Calle de la Maestranza, 9 - 15001, A Coruña, Spain; c Servicio de Ginecología, Hospital Universitario Lucus Augusti (HULA). Servizo Galego de Saúde (SERGAS), Spain

**Keywords:** Bacterial vaginosis, Microbiome, Machine learning, Consensus clustering analysis, Treatment response

## Abstract

This study aimed to develop a robust classification scheme for stratifying patients based on vaginal microbiome. By employing consensus clustering analysis, we identified four distinct clusters using a cohort that includes individuals diagnosed with Bacterial Vaginosis (BV) as well as control participants, each characterized by unique patterns of microbiome species abundances. Notably, the consistent distribution of these clusters was observed across multiple external cohorts, such as SRA022855, SRA051298, PRJNA208535, PRJNA797778, and PRJNA302078 obtained from public repositories, demonstrating the generalizability of our findings. We further trained an elastic net model to predict these clusters, and its performance was evaluated in various external cohorts. Moreover, we developed VIBES, a user-friendly R package that encapsulates the model for convenient implementation and enables easy predictions on new data. Remarkably, we explored the applicability of this new classification scheme in providing valuable insights into disease progression, treatment response, and potential clinical outcomes in BV patients. Specifically, we demonstrated that the combined output of VIBES and VALENCIA scores could effectively predict the response to metronidazole antibiotic treatment in BV patients. Therefore, this study's outcomes contribute to our understanding of BV heterogeneity and lay the groundwork for personalized approaches to BV management and treatment selection.

## Highlights

• VIBES predicts robust subtypes of the vaginal microbiome based on minimal set of species.
• The study introduces VIBES, a tool designed to facilitate the prediction of new clusters in external cohorts.
• VIBES offers valuable insights into the clinical implications of microbiome subtypes, such as BV diagnosis and treatment response.

## Graphical abstract

**Figure:**
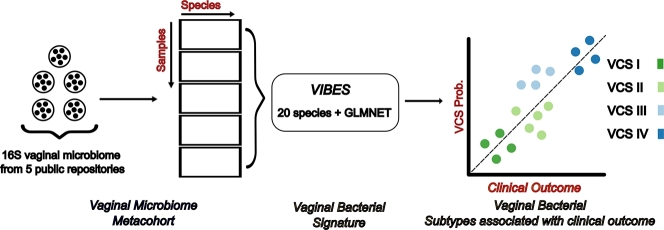

## 1 Introduction

The human vagina is a balanced and dynamic ecosystem, with a complex population of aerobic and anaerobic bacteria, which can reach up to 10^9^ cfu/mL of vaginal fluid [41]. Vaginal microbiota is considered healthy when the bacteria present inside the vagina comprise 90-95% *Lactobacillus spp*. However, other studies indicate that it cannot be used as the sole criterion for establishing vaginal health [9].

Bacterial vaginosis is an underdiagnosed disease characterized by a heterogeneous group of individuals with distinct phenotypes and varying responses to treatment [21], [33]. This heterogeneity has prompted the exploration of alternative classification criteria to stratify patients and obtain more specific information about the condition [1], [23], [42], [20].

Previous studies have demonstrated the usefulness of using bacterial species as predictive signatures for different diseases, such as pancreatic cancer [15], diabetes [10] or inflammatory bowel disease [17]. Motivated by these findings, our objective was to establish a robust, simple and explainable signature of bacterial species that could effectively categorize patients into different groups. Furthermore, we developed VIBES an user-friendly R package that encapsulates the software for convenient implementation.

The reduced diversity of vaginal microbiome simplifies the characterization, interpretation and identification of key microbial players and their potential impact on health and disease. To evaluate the efficacy of our model, we compared its performance with VALENCIA community state type (CSTs) [12], an existing and well-established classification criteria. While there are shared information between the two approaches, our model provided distinct insights into treatment response of metronidazole antibiotic.

Both VALENCIA and VIBES are two models generated through different approaches that could complementarily explore the vaginal microbiome. Broadly, the main differences that have led to proposing the complementary use of both in terms of features, underlying algorithms, taxonomic coverage, and class structure are the following: VALENCIA, a model incorporating up to 199 features, operates on the K-Nearest Neighbors (KNN) algorithm, while VIBES, on the other hand, utilizes the Elastic Net (glmnet) algorithm and encompasses 20 features. One notable difference between the two models is their taxonomic coverage: VALENCIA incorporates taxa ranging from the class level down to the species level, while VIBES solely considers species-level taxonomic information. Moreover, VALENCIA presents a class structure consisting of five classes, which can further be subdivided into up to thirteen subclasses, whereas VIBES features four distinct classes. By combining the strengths of both approaches, we aimed to enhance the accuracy and comprehensiveness of predictions related to treatment response and other clinical factors.

Our study objectives encompassed three main aspects. Firstly, we aimed to introduce a novel clustering approach to stratify BV patients based on their microbiome profiles to provide additional information to the VALENCIA. This clustering analysis would help identify distinct patient groups and facilitate targeted interventions. Secondly, we sought to assess the generalizability and robustness of our ML model by evaluating its performance across multiple external cohorts. Finally, we aimed to compare the predictive capacity of our classification system in terms of treatment responses, further highlighting the value of our model in clinical decision-making.

## 2 Results

Introducing the findings of our scientific inquiry, this article unfolds in three distinct sections, each contributing to a comprehensive understanding of our research outcomes. In the first section, we delve into the results of consensus clustering, unveiling four clusters that exhibit both generalizability and robustness across diverse cohorts. Subsequently, we explore the intricacies of VIBES, an explainable, robust, and generalizable machine learning model, in the second section, shedding light on its capacity to predict the identified consensus clusters. The third section of our presentation focuses on a pivotal aspect of our investigation: the enhancement of treatment response prediction to metronidazole. Together, these sections form a cohesive narrative, elucidating the multifaceted dimensions of our study and providing a nuanced perspective on the intricacies of our findings.

### 2.1 Consensus clustering identifies four generalizable and robust clusters across cohorts

In this study, we used consensus clustering analysis to stratify patients with BV and healthy individuals, based on the microbiome profiles of their vaginal flora. To ensure a robust and generalizable stratification criterion, we selected 22 species that were shared across all cohorts. This strategy effectively minimized the potential for overfitting to the discovery cohort, allowing for reliable extrapolation of the identified clusters to the other cohorts.

The analysis revealed the presence of four distinct groupings within the examined population. We explored various combinations of data transformations (counts, ALR, and CLR), distance methods (Pearson, Spearman, Euclidean, binary, and maximum), and clustering algorithms (hierarchical clustering, partitioning around medoids, and k-means). After careful consideration, we identified the most optimal configuration, involving CLR-transformed data, the Euclidean distance function (reflecting Aitchison distance), and k-means clustering. This particular configuration, consisting of 4 robust and stable clusters across multiple iterations, was determined as the most suitable. The number of clusters (4) was rigorously established using the *ConsensusClusterPlus* R package, employing a consensus clustering approach to assess stability and robustness. This method ensures a reliable determination of *K*, supported by the consensus matrix and cumulative distribution function (CDF) plot, as illustrated in Supplementary Figure S1.

As we mentioned earlier, to assess the robustness of the clusters, we conducted a bootstrap analysis using different subsamples. When comparing the similarity of the clusters in each of these subsamplings to the original clusters (using all the samples), we obtained Adjusted Rand Index (ARI) values of 0.875 with 50% of the data, 0.878 with 62.5%, 0.935 with 75%, and 0.965 with 90% of the data. This yielded an average ARI value of 0.914. The findings suggest that the optimal number of four clusters appears to be determined more by the nature of the problem than by the number of samples included in the CCP.

An essential aspect of validating the robustness and generalizability of our clustering analysis is to examine the distribution of our identified clusters across multiple external cohorts. By assessing the consistency of cluster assignments in independent datasets, we can ascertain the reliability and reproducibility of our findings.

**Fig. 1:**
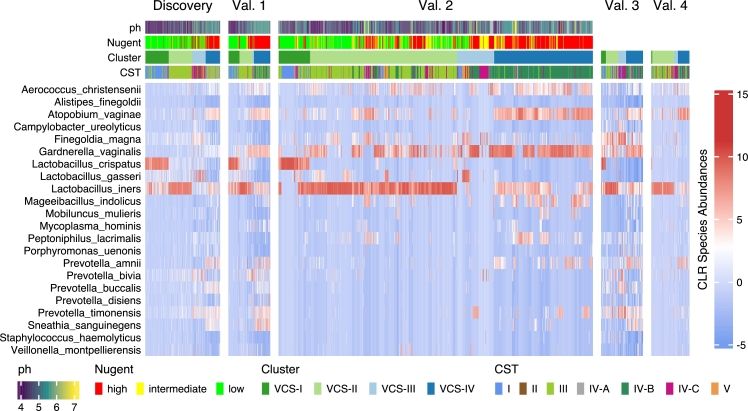
Heatmap of the 22 species across the 5 cohorts. The pH, the Nugent Score, the clusters generated and the VALENCIA CSTs are shown for each sample.

Notably, we observed in Fig. 1 a remarkable similarity in the distribution of our clusters across diverse external cohorts. This consistency reinforces the reliability of our clustering approach and suggests that the identified clusters are not specific to our study population alone but rather reflect inherent characteristics of BV.

Specifically, VCS-I exhibited a predominance of *Lactobacillus* species, with higher abundances of *Lactobacillus crispatus* and *Lactobacillus iners* compared to the other clusters. The difference in abundance of *Lactobacillus crispatus* is significant. This cluster was characterized by a relatively balanced microbiome composition, resembling a healthy vaginal microbiota.

VCS-II displayed a higher abundance of *Lactobacillus iners*, without presence of *Lactobacillus crispatus*.

VCS-III demonstrated a significantly higher abundance in another *Lactobacillus* species such as *Lactobacillus gasseri*. These patients displayed a distinctive microbiome profile, suggestive of an altered vaginal environment.

VCS-IV displayed a higher abundance of *Gardnerella vaginalis*, *Prevotella* species, *Sneathia Sanguinegens* and *Atopobium vaginae*, indicating dysbiosis and a shift towards a more pathogenic microbiome.

To further assess the validity and uniqueness of our clustering approach, in Fig. 1 we also compared our identified clusters with the subsets defined by the VALENCIA classification method and Nugent score.

The Nugent score categorized patients based on their clinical manifestations and symptomatology, focusing on the clinical presentation of BV rather than the underlying microbiome composition. In contrast, our clustering approach was primarily driven by the microbiome species abundances, allowing for a more granular understanding of the vaginal ecosystem.

Interestingly, we found that some subsets within the VALENCIA CSTs could be mapped to specific clusters in our analysis. For example, patients exhibiting the asymptomatic subset in VALENCIA corresponded predominantly to VCS-I in our clustering analysis. This concordance suggests that patients within this subset tend to possess a healthier vaginal microbiota characterized by higher abundances of *Lactobacillus species*, specifically with presence of *Lactobacillus crispatus*.

However, there were notable differences between the two classification systems as well. For instance, we observed that certain VALENCIA CSTs (CST-III, CST-IV-A, and CST-V) did not align directly with any specific cluster in our analysis. Specifically, intermediate clusters from VIBES such as VCS-II and VCS-III show high heterogeneity when compared with CST subtypes. This discrepancy indicates that our clustering approach provides additional and complementary insights into the microbiome-based characteristics of BV patients.

In the subsequent sections, we will further explore the characteristics and clinical implications of each cluster, as well as evaluate the performance of our ML model in predicting treatment responses within these distinct groups.

### 2.2 VIBES as an explainable, robust and generalizable machine learning model to predict consensus clusters

Building upon the previous cluster analysis, we developed VIBES an elastic net model to predict clusters of BV patients. This approach capitalizes on the insights gained from earlier cluster analyses and leverages the predictive power of ML techniques to enhance the accuracy and applicability of BV cluster prediction.

By integrating the knowledge derived from previous cluster analyses, our ML model benefits from the identification of meaningful patterns and relationships among BV patients. This enables the model to capture the intricate interplay of variables and features that differentiate the various clusters.

**Fig. 2:**
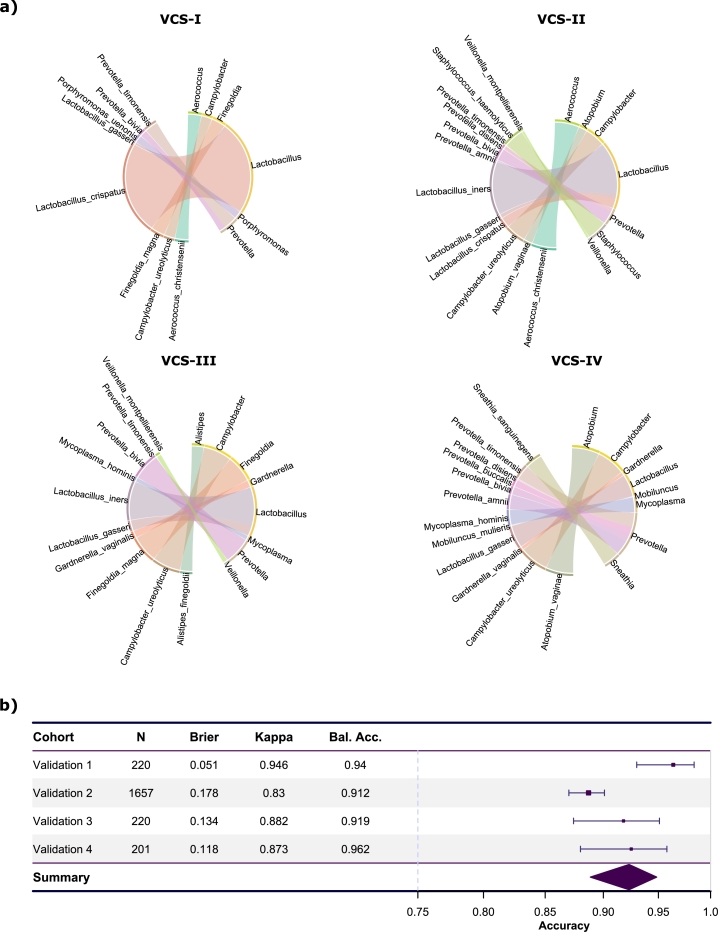
Variable importance and external validation summary using VIBES. **a)** The betas assigned to each species (and their representation by genus) for each cluster-specific model (VCS-I to VCS-IV). **b)** Performance measures across the four validation cohorts.

In order to gain insights into the important features driving the model's predictions, we analyzed the variable importance using beta values. The beta values in the elastic net model refer to the coefficients assigned to each predictor variable and represent the weights/strengths assigned to each predictor variable in the final model. Fig. 2a displays the variable importance of each linear model specific by cluster, providing an interpretability aspect to our model. In order to assess the significance of the microbial balance in the vaginal microbiome, we organized the weights of the beta coefficients into groups based on the genus classification. This approach allowed us to evaluate the relative importance of different microbial taxa in shaping the composition of the vaginal microbiota. By grouping the beta weights, we gained insights into the contributions and potential roles of specific microbial genera in maintaining a balanced vaginal ecosystem, contributing to BV cluster predictions. Thus, Fig. 2a and Supplementary Figure S2 depict the beta coefficients obtained from the elastic net model for the 20 species encompassing all four clusters.

Initially, it should be noted that two species, namely *Mageeibacillus indolicus* and *Peptoniphilus lacrimalis*, are excluded from the visual representation due to their beta coefficients being consistently zero across all clusters. This implies that these species do not contribute any significant information to the model and have no discernible impact on the observed clustering patterns. The analysis reveals that within VCS-I, the species exhibiting the highest beta value is *Lactobacillus crispatus*, suggesting a robust association between this species and the specific cluster. Moving to VCS-II, the species *Lactobacillus iners*, *Aerococcus christensenii*, *Campylobacter ureolyticus*, and *Staphylococcus haemolyticus* exhibit the highest beta values, underscoring their importance in predicting this class. Similarly, in VCS-III, the most influential species are *Lactobacillus iners*, *Campylobacter ureolyticus*, *Prevotella bivia*, and *Finegoldia magna*. Lastly, VCS-IV presents the most variability in terms of importance. *Atopobium vaginae*, *Campylobacter ureolyticus*, *Lactobacillus gasseri*, *Mycoplasma hominis*, and *Sneathia sanguinegens* obtained the highest beta values in the model. These findings offer valuable insights into the discriminatory features of the dataset by revealing the specific species that drive the classification of each cluster, providing a deeper understanding of the problem.

To assess the performance of our model, we conducted external validations on four separate cohorts. Fig. 2b presents the performance metrics, including the Brierś error, Cohenś kappa statistic, accuracy, and balanced accuracy, for each cohort. It can be seen that having optimized the hyperparameter configuration, the values of all measurements improve compared to the first external cross-validation (see Supplementary data Figure S3). During the validation of the elastic net model, high accuracy values (> 0.91) were achieved, except for the validation cohort 2, which showed a slightly lower accuracy of approximately 0.87. Furthermore, the Brier error in this particular cohort was slightly higher compared to the other cohorts, with an approximate value of 0.18. This performance difference may be due to the difference in the microbiome profile of this cohort compared to the rest of the cohorts. Nonetheless, all cohorts demonstrated notably high values of balanced accuracy, surpassing 0.91. In addition, Cohen's kappa was introduced for this validation, which yields very good values (> 0.83). The results obtained were highly promising, indicating the model's effectiveness in predicting BV clusters across different datasets. The observed metrics consistently demonstrated excellent performance, suggesting the robustness of our model in classifying BV patients subtypes.

Alongside model development, we created an R package to streamline the utilization of our model for researchers and practitioners in the field. This package has been designed to simplify the usage of our model, and it comes with user-friendly functions and comprehensive documentation. We have made sure that it is accessible and convenient for a wide range of users. As it is developed in a user-friendly way, it uses the base packages that come with R installed as a base. The only dependency is on the *phyloseq* [19] package to be able to incorporate the objects that this package produces. VIBES utilizes the best-performing model and incorporate a key microbiomics-based signature of selected microorganisms. This package, accepts a matrix, *dataframe*, or *phyloseq* object containing a vaginal microbiome profile as input. From this profile it returns the probabilities (between 0 and 1) of belonging to each of our clusters (VCS-I, VCS-II, VCS-III or VCS-IV) and by consensus to which one it belongs. This output variables could be used in downstream analysis or in other predictive models.

The documentation for the VIBES package, along with a use case, is available at https://mall-machine-learning-in-live-sciences.github.io/VIBES-docs/.

Overall, our proposal demonstrated strong predictive performance in identifying BV clusters. The variable importance analysis enhanced the interpretability of the model, aiding in the understanding of the key factors driving the predictions. Furthermore, the development of an easy-to-use R package aims to facilitate the adoption and application of our model in clinical and research settings.

### 2.3 Improving the prediction of treatment response to metronidazole

**Fig. 3:**
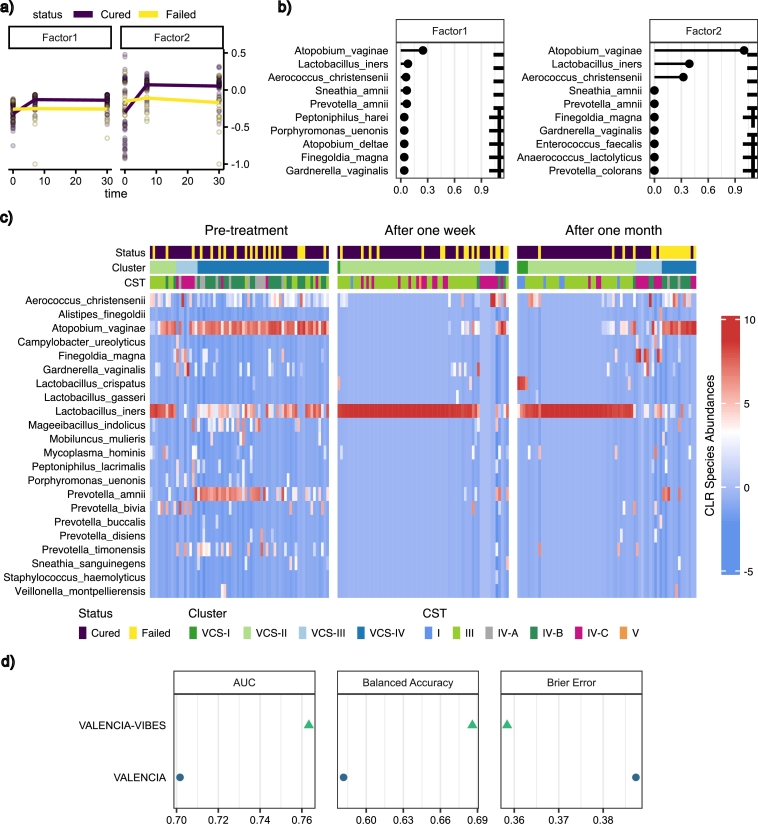
Changes in microbiome profiles after treatment with metronidazole. **a)** illustrates the variance explained by the extracted factors. In **b)** show the weight that each factor gives to the species. **c)** represents the heatmap with the abundance of the 22 species over the three-time points (pre-treatment, after one week, and after one month) regarding metronidazole treatment. In this case, the cluster labels have been obtained using VIBES. **d)** shows the prediction performance (RF algorithm and mean of the 10 fold-CV) of the VALENCIA CSTs, and with VIBES in relation to the response to metronidazole.

To assess the utility of our model in predicting treatment response, we conducted a longitudinal cohort study consisting of three-time points: pre-treatment, after one week, and after one month. The analysis involved the evaluation of microbiome profiles using the MEFISTO method, capturing the changes in vaginal microbiota following treatment. The results, depicted in Fig. 3a-b, highlight the dynamic alterations observed in the microbiome profiles of responders compared to non-responders.

Fig. 3a present the results of the MEFISTO analysis, illustrating the shifts in the vaginal microbiome profiles after treatment. Non-responders exhibited minimal changes in their microbiome profiles, indicating a lack of response to the treatment. In contrast, responders demonstrated notable alterations, indicating a successful response to the intervention.

Building upon the encouraging findings from our previous results, we aimed to explore the relationship between the clusters and the various timepoints. By investigating the temporal dynamics of the clusters, we sought to gain a deeper understanding of their behavior and potential implications across different stages or intervals of the study. Fig. 3c displays a heatmap of the microbiome profiles across the three-time points. The samples were ordered based on the probabilities assigned by our model, and they were labeled with both VALENCIA CSTs and treatment response status. The heatmap uncovers a discernible association between treatment responders and non-responders with the clusters we identified. It can be seen that at baseline most patients have a microbiome profile with dysbiosis, few *Lactobacillus* species (except for some patients with *Lactobacillus iners*), an abundance of *Atopobium vaginae*, and *Prevotella amnii*. One week after antibiotic ingestion, dysbiosis was observed to have disappeared in most patients, leaving a predominance of *Lactobacillus iners*. At the end-point, patients who did not respond to treatment showed incipient dysbiosis. With regard to stratification, most of the patients who did not recover corresponded to cluster VCS-IV and to a lesser extent to VCS-III. This finding highlights the promising capability of our model and clusters to capture unique microbiome patterns that are linked to treatment response, suggesting their potential as valuable indicators in this context.

Based on this premise, we formulated a hypothesis that the variables obtained from the mentioned package could serve as predictive variables for a new prediction model. Thus, we conducted a benchmark analysis to predict the response to metronidazole antibiotic in pretreated patients. Predictors were employed in three distinct combinations of variables, namely VALENCIA, VIBES, and their composite. The results of this experiment, as shown in Fig. 3d, revealed an intriguing finding. Remarkably, the combined use of VALENCIA and VIBES variables outperformed the individual approaches. This noteworthy outcome suggests that our identified clusters offer unique and complementary information beyond what can be captured by VALENCIA alone. Consequently, our clusters present valuable insights for predicting treatment response, thereby enhancing clinical decision-making.

## 3 Methods

All experiments have been carried out in R (4.0.2 version) [27].

### 3.1 Datasets

To carry out this work, five different cohorts have been used. All of them correspond to 16S rRNA sequencing samples obtained from vaginal environment. SRA022855 (discovery cohort) [31] has been used to compute the reference clusters, as well as to train the ML models. The remaining cohorts, SRA051298 (validation cohort 1) [38], PRJNA208535 (validation cohort 2) [30], PRJNA797778 (validation cohort 3) [11], and PRJNA302078 (validation cohort 4) [45], have been used to validate the results. Moreover, we used the validation cohort 4 to explore the relationship between our stratification methodology and the response to metronidazole antibiotic.

In this context, the study employed microbial data from various public repositories, incorporating information from diverse cohorts: SRA022855 featured the vaginal bacterial communities of 396 North American women (Baltimore and Atlanta), aged 12 to 45 (mean age: 30), with participants self-identifying as African American (n=104), White (n=98), Hispanic (n=97), and Asian (n=97). SRA051298 included data from 220 North American women (Seattle), aged 18 to 57 (mean age: 29), self-identifying as African American (n=75), White (n=97), Asian (n=15), and Others (n=33). PRJNA208535 involved the vaginal bacterial communities of 25 North American women (Birmingham) over 10 weeks (1657 16S raw samples on the repository), aged 19 to 45 (mean age: 27), with participants self-identifying as African American (n=20) and White (n=5). PRJNA797778 focused on the vaginal bacterial communities of 39 North American women (Baltimore) over 10 weeks (220 16S raw samples on the repository), aged 19 to 45, with participants self-identifying as African American (n=24), White (n=10), Hispanic (n=4), and Asian (n=1). PRJNA302078 examined the vaginal bacterial communities of 65 Chinese women (Beijing) at three time points: pretreatment, one week after treatment, and one month after treatment with metronidazole (201 16S raw samples on the repository), aged 18 to 53.

The cohorts were obtained by processing the 16S rRNA sequences hosted in the European Nucleotide Archive (ENA). The samples were downloaded from ENA using the *enaBrowserTools*. In the case of the validation cohort 2, we only downloaded those with available clinical information. Subsequently, the *DADA2* [6] package was used to process these samples. Non-chimeric assembled amplicon sequence variants (ASVs) were taxonomically assigned (phylum to species) using the Silva reference database (v138.1) [18].

The discovery cohort was obtained from [40], while the validation cohort 1 was sourced from [38]. The downloaded data from both cohorts included clinical information and count tables for Operational Taxonomic Units (OTUs). Unfortunately, the ENA did not provide reliable access to demultiplexed samples or their identification. As an alternative, we used the OTU names and accessed the Taxonomy database [34] to acquire the associated taxonomic categories. For this purpose, we used: *rentrez* [44], *taxonomizr* [37] and *taxize* [35].

OTU/ASV tables, taxonomic table, and clinical data of the patients were combined in a *phyloseq* [19] object. Finally, each *phyloseq* was aggregated at the species level, and species not present in all cohorts were filtered out to perform the downstream analysis, resulting in 22 common species.

### 3.2 Cluster analysis

For the analysis of patient data in the discovery cohort, the *ConsensusClusterPlus* (CCP) method [43] was employed in this study. The following parameters were used: a maximum of 6 clusters, 1500 resampling iterations, a resampling threshold of 0.8 without resampling of features, and a comprehensive evaluation of all available distance measures compatible with the clustering algorithm.

Furthermore, we conducted experiments using three distinct data transformation methods (counts, ALR, and CLR) and three clustering algorithms (hierarchical clustering, partitioning around medoids, and k-means).

Additionally, a bootstrap analysis was performed alongside the CCP method to enhance the robustness and reliability of our clustering results. This process involved creating four distinct subsets by subsampling data, which covered 50%, 62.5%, 75%, and 90% of the original dataset. Subsequently, we applied the CCP method to each of these subsets, allowing us to assess the stability and consistency of our clustering results in different subsamples. To quantify the similarity of the resulting clusters from each subset of samples with respect to the clusters with all the samples, the Adjusted Rand Index (ARI) was used. This index measures the agreement between two sets of clusters and provides information about the robustness of the clustering solution. The *mclust* package [36] was utilized for calculating this index.

### 3.3 Supervised classification

We used *mlr3* (0.14.1 version) R package [16] to train all ML models. Specifically, in the benchmark analysis we used four different algorithms: Random Forest (RF) [5], Support Vector Machines (SVM) [8], Elastic Net (glmnet) [13], and eXtreme Gradient Boosting (xGBoost) [7].

A nested cross-validation was performed for training the algorithms using the discovery cohort. This type of validation consists of two CV processes: the first is an independent internal CV (a holdout of 0.80 for training and 0.20 for validation) for the selection of the best hyperparameters of each algorithm, and the second is an independent external CV (10-fold CV) to evaluate the model in general. Once all the models had been trained on the discovery cohort, their performance was evaluated on the validation cohorts.

For the evaluation of the models, the following measures have been calculated: accuracy (Acc), balanced accuracy (B.Acc), Brier error (Brier), and Cohenś kappa coefficient (Kappa).

### 3.4 Longitudinal analysis

Furthermore, considering that validation cohort 4 exhibited a response to BV treatment, our aim was to investigate the dynamics of these clusters across multiple treatment time points. To gain comprehensive insights, we employed the *MOFA2* package [3] and conducted a MEFISTO analysis. This analysis allowed us to extract valuable information pertaining to the treatment response, including the identification of species that played a pivotal role and their potential associations with the newly discovered clusters. The MEFISTO pipeline enabled us to explore and uncover important patterns and relationships within the data.

## 4 Discussion

Our study employed the consensus cluster approach to identify distinct clusters within the dataset. The clustering analysis revealed four clusters that exhibited robust and stable patterns across multiple iterations and large external cohorts. These clusters represented different subgroups within the data, highlighting the presence of significant heterogeneity. This finding is consistent with previous studies that have demonstrated the existence of diverse microbial communities within similar populations [12].

After performing the cluster analysis, we proceeded to develop a ML model to predict the identified clusters. This step aimed to provide a practical and efficient tool for assigning new samples to the appropriate cluster based on their characteristics. The ML model was trained using a diverse set of features that exhibited significant discriminatory power in distinguishing between the clusters.

To ensure the accessibility and usability of our approach, we further developed an R package, which encapsulates the predictive model and offers a user-friendly interface for researchers and practitioners. The package, named VIBES, provides a comprehensive set of functions and tools for data preprocessing and cluster prediction. Its modular structure allows for easy integration into existing workflows, facilitating seamless implementation in various research and clinical settings.

The predictive performance of our ML model was rigorously evaluated using appropriate metrics, such as accuracy, balanced accuracy, kappa, and brier error. Cross-validation techniques were employed to assess the robustness of the model and guard against overfitting. Additionally, external validation using independent datasets would help confirm the generalizability and reliability of our approach.

Notably, the variable importance analysis using the elastic net model provided valuable insights into the predictive power of specific variables for each class. Within VCS-I, *Lactobacillus crispatus* is likely to exhibit a prominent role, exerting a substancial influence on the overall microbial composition, and suggesting its potential role as a key indicator for this class. *Lactobacillus crispatus* is considered a dominant bacterium in a healthy vaginal microbiota and plays a protective role in maintaining microbial balance [32], [24], [29]. It produces lactic acid as a byproduct of its metabolism. This metabolic trait contributes to the maintenance of an acidic vaginal environment, which serves as a natural defense mechanism against the proliferation of pathogenic bacteria, thereby preserving vaginal health. In contrast, as the analysis moves towards VCS-IV, the presence and impact of *Lactobacillus crispatus* appear to diminish, suggesting a lesser contribution to the overall microbial dynamics.

Similarly, VCS-II, VCS-III, and VCS-IV, revealed distinct sets of variables that exhibited high beta values, emphasizing their importance in predicting their respective classes.

In VCS-II and VCS-III, *Lactobacillus iners* appear to be predominant, while it is absent in VCS-I and VCS-IV. Despite the general notion of a protective effect associated with *lactobacilli*, there is controversy surrounding *iners*, which has been identified in diseased vaginal samples [14], [26].

*Prevotella* is known to produce putrescine, cadaverine, and trimethylamine, which can contribute to an increase in vaginal pH. Additionally, *Prevotella* produces ammonium, creating an environment that favors the growth of Gardnerella, as observed in VCS-III and VCS-IV.

The production of putrescine, cadaverine, and trimethylamine by *Prevotella* highlights its potential role in altering the vaginal microenvironment. These metabolites are associated with an increase in vaginal pH, which can disrupt the delicate balance of the vaginal ecosystem and contribute to the development of dysbiosis or vaginal disorders. Furthermore, *Prevotella bivia* is known to produce enzymes and toxins that can contribute to tissue damage and inflammation. It can also interact with other bacteria and host factors, further exacerbating the infection or disease progression [22], [21], [25], [28].

In VCS-IV, elevated beta values of the bacterial species *Atopobium vaginae* and *Sneathia sanguinegens* were observed suggesting a potential association between these bacterial species and the underlying factors driving the microbial community dynamics in this particular cluster. *Atopobium vaginae* is a gram-positive bacterium that produces enzymes and metabolites that can disrupt the delicate balance of the vaginal ecosystem and contribute to the development of symptoms associated with BV, leucorrhea, high pH, and the presence of clue cells. It is especially associated with the formation of a biofilm that favors resistance to metronidazole and recurrences [4], [20], [22], [21], as depicted in Fig. 2. *Sneathia sanguinegens*, formerly known as *Leptotrichia sanguinegens*, is another gram-negative bacterium associated with various gynecological infections, and has been found to produce virulence factors that can contribute to inflammation and tissue damage in the vaginal environment [2], [21], [33], [39]. Finally, elevated beta values are observed for *Campylobacter ureolyticus* and *Mycoplasma hominis*, which appears to be associated with an increase in *Lactobacillus gasseri*. This finding suggests a potential interplay between these bacterial species within the vaginal microbiota. *Campylobacter ureolyticus* and *Mycoplasma hominis* are known microbial inhabitants of the vaginal ecosystem. Previous studies have implicated these bacteria in various aspects of vaginal health and microbial dysbiosis. The elevated beta values observed in VCS-IV may reflect an ecological shift or dysregulation in the vaginal microbial community. This dysbiosis could potentially disrupt the delicate balance of beneficial and pathogenic microorganisms. Consequently, the increased abundance of *Campylobacter ureolyticus* and *Mycoplasma hominis* may create an environment conducive to the proliferation of *Lactobacillus gasseri*. *Lactobacillus gasseri*, known for its probiotic properties and beneficial effects on vaginal health, may respond to the changes in the microbial composition induced by *Campylobacter ureolyticus* and *Mycoplasma hominis*. It is possible that *Lactobacillus gasseri*, as a competitive and resilient bacterium, increases in abundance as a defense mechanism to restore the vaginal microbiota homeostasis and counteract the potential negative effects associated with the presence of *Campylobacter ureolyticus* and *Mycoplasma hominis*.

Our findings align with prior research, which has implicated these variables in various aspects of microbial community composition and health outcomes.

It is important to acknowledge the limitations of this study. First, the dataset used in our analysis was derived from a specific population, which may limit the generalizability of our findings to other populations or geographical regions. Furthermore, we selected the discovery cohort with the largest population of patients to enhance the generalizability of our findings and ensure the robustness of the identified subtypes. It is important to note that the taxonomic assignment in this dataset was based on OTUs, which may not be the most precise method. However, the results obtained from external datasets, where ASVs were used for taxonomic assignment, validate the robustness of the relationships observed in our dataset. This validation across different datasets suggests that our findings hold substantial strength and can be reliably applied beyond the initial cohort. Additionally, although the consensus clusters and elastic net models are widely used and have shown robust performance, they are not without their own inherent biases and assumptions. Future studies should aim to validate our findings using independent datasets and alternative clustering algorithms to confirm the stability and reproducibility of the identified clusters.

## 5 Conclusion

In conclusion, this paper introduces a novel approach for predicting vaginal microbiome subtypes using explainable machine learning models. By precisely identifying and categorizing these subtypes, a deeper understanding of microbial diversity within the vaginal ecosystem is achieved. The findings significantly contribute to our comprehension of the complex nature of the vaginal microbiome and its potential implications for health and disease. Moreover, the ability to predict subtypes opens avenues for personalized interventions and targeted therapies based on specific microbial profiles. The research holds promise for enhancing our understanding of the vaginal microbiome and contributing to improved health outcomes. Future studies can build upon these findings to further explore the functional implications of subtypes and develop interventions that leverage the individualized characteristics of the vaginal microbiome.

## Funding

CITIC is funded by the 10.13039/501100010801Xunta de Galicia through the collaboration agreement between the Ministry of Culture, Education, Vocational Training, and Universities and the Galician universities for the strengthening of research centers in the University System of Galicia (CIGUS). The authors acknowledge the support of CESGA (Centro de Supercomputación de Galicia) for providing computing resources and related technical support that contributed to the research results reported in this paper. JLB work was financed by the Spanish 10.13039/501100023561Ministry of Universities by means of the Margarita Salas (RSUC.UDC.MS06) linked to the European Union through the NextGenerationEU program.

## Availability of data and materials

The bacterial 16S rRNA gene sequences from all cohorts (SRA022855, SRA051298, PRJNA208535, PRJNA797778, and PRJNA302078) can be accessed via their respective accession numbers on the ENA Browser.

## Code availability

The source code to reproduce all the analysis, along with documentation, is available on GitHub: https://github.com/MALL-Machine-Learning-in-Live-Sciences/BV_Microbiome.

VIBES can be downloaded and installed directly from: https://github.com/MALL-Machine-Learning-in-Live-Sciences/VIBES.

## CRediT authorship contribution statement

**Diego Fernández-Edreira:** Data curation, Writing - Original draft preparation, Software. **Jose Liñares-Blanco:** Conceptualization of this study, Data curation, Methodology, Writing - Original draft preparation. **Patricia V.-del-Río:** Conceptualization of this study, Writing - Original draft preparation. **Carlos Fernandez-Lozano:** Conceptualization of this study, Methodology, Writing - Original draft preparation.

## Declaration of Competing Interest

Authors declare no conflict of interest.

## Appendix A Supplementary material

MMC Machine learning-based consensus subtyping of the vaginal microbiome. Supplementary Figures. MMC
