# Supplementary material for: VIBES: A consensus subtyping of the vaginal microbiota reveals novel classification criteria
Source: Comput Struct Biotechnol J. 2023 Nov 30;23:148–56. doi: 10.1016/j.csbj.2023.11.050 (PMC10749217; doi:10.1016/j.csbj.2023.11.050)
Supplement: MMC — Machine learning-based consensus subtyping of the vaginal microbiome. Supplementary Figures. [file mmc1.pdf]

Supplementary Figures

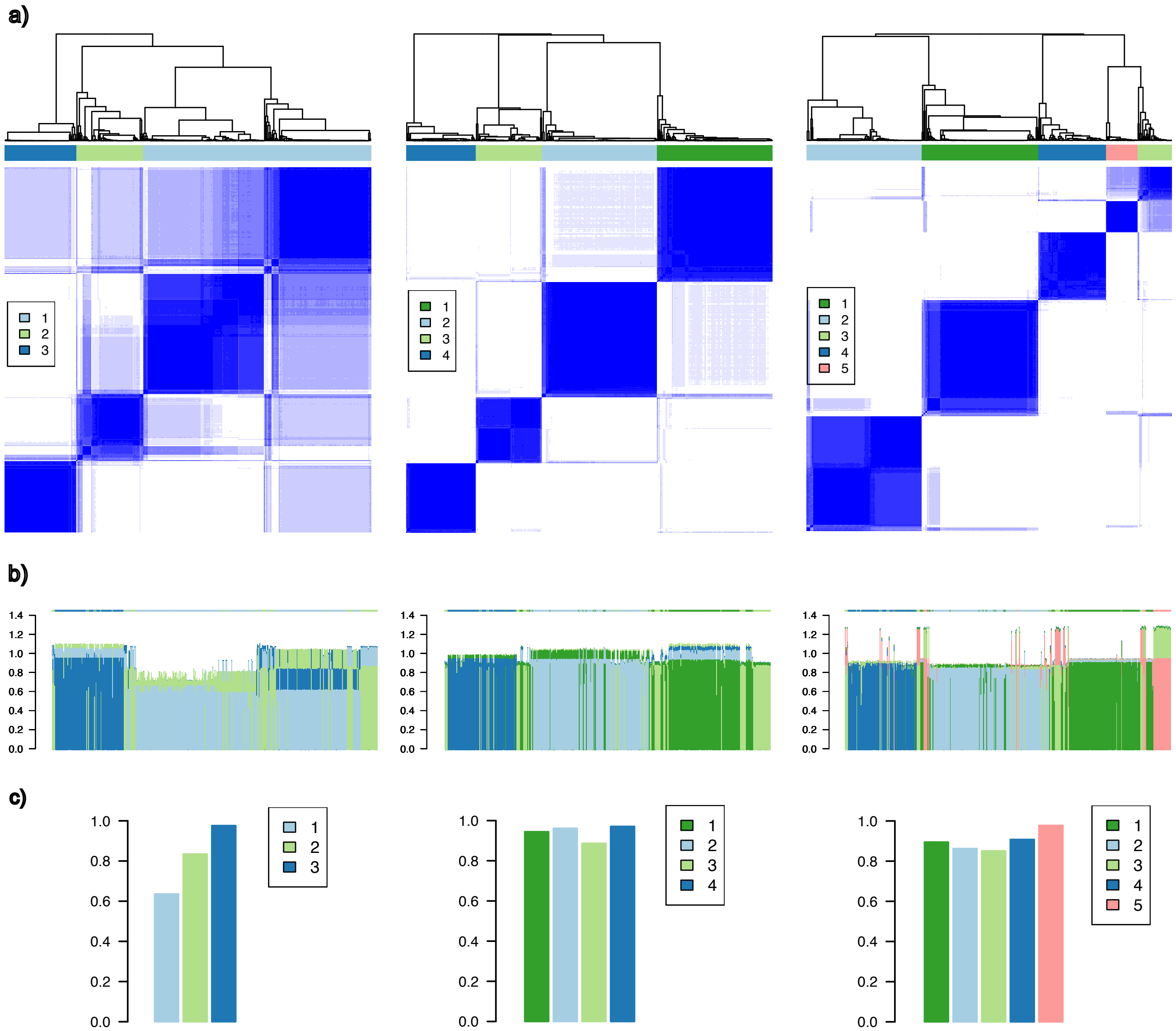

Figure 1: ConsensusClusterPlus plots. **a)** shows the consensus matrices where the samples are arranged in rows and columns. The intensity shows the probability of clustering together, where white is 0 and dark blue is 1. It can be observed that the intensity of these four clusters is higher than that produced by analyses establishing a number of clusters of three or five. **b)** displays the consensus of each sample's assignment to a particular cluster for a given number of k clusters. The higher the bar for a particular cluster, the greater the consensus that the sample belongs to that cluster. In this case, the consensus values for the clusters when k = 4 are more unified, and there is less discordance, indicating a tendency towards greater purity. **c)** shows the cluster-consensus value of clusters at each k. High values indicate a cluster has high stability and low values indicate a cluster has low stability. In this case, the clusters made with a k = 4 obtain high mean consensus values (> 0.85) as well as less dispersion between the different values of each cluster.

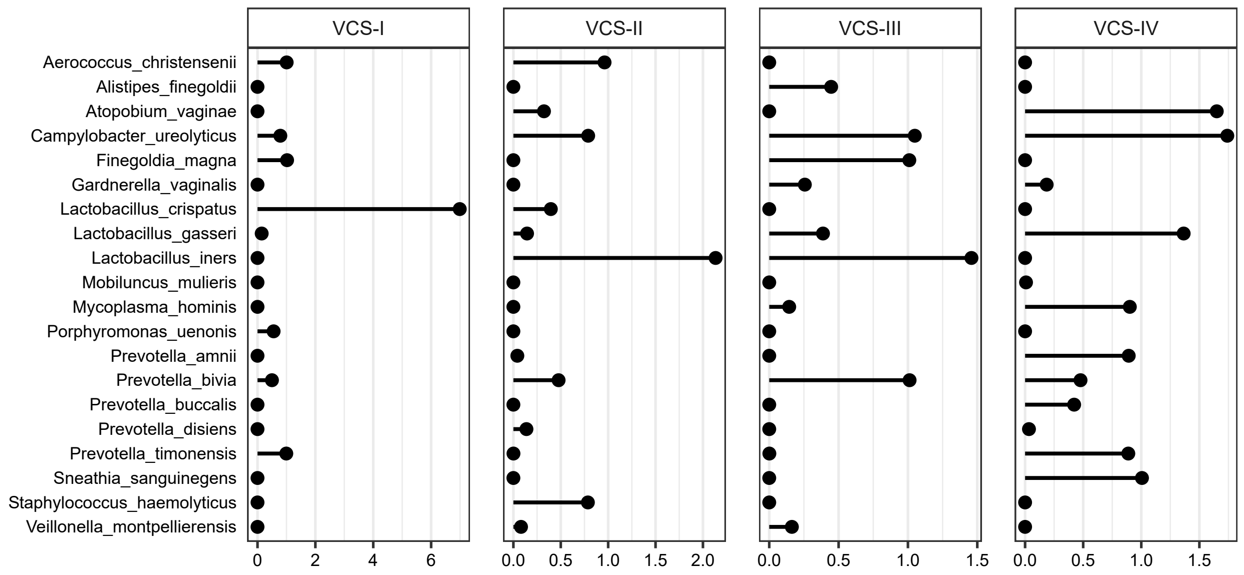

Figure 2: Display of the betas that the model used by VIBES assigns to each species

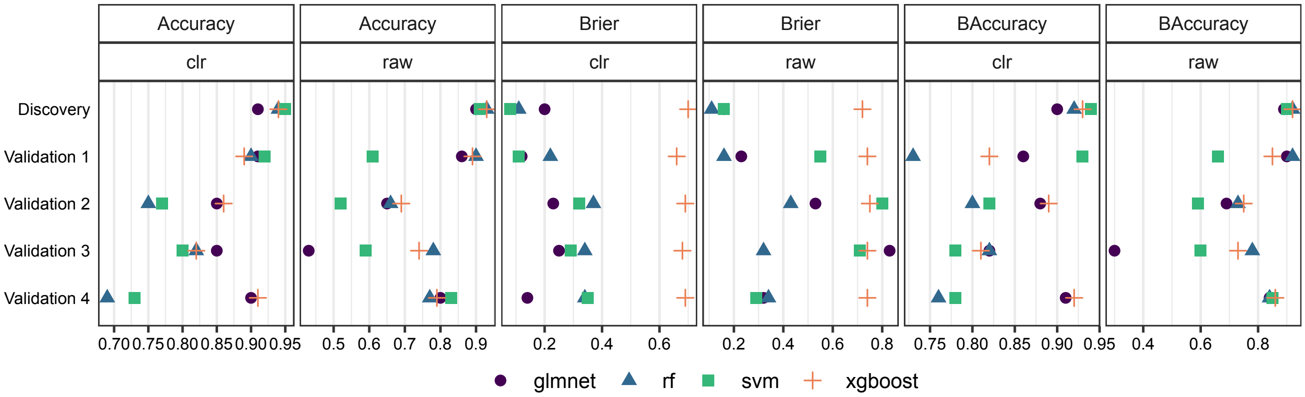

Figure 3: Benchmark experiments with the four algorithms tested. Performance has been measured in duplicate: using the counts and the CLR transformation. Regarding accuracy, using the CLR-transformed data yields higher performances in all cohorts. Although using counts some similar performance can be achieved in global computation, they have lower values and present much more dispersion. Looking at the Brier error, it can be seen that xGBoost reaches very high values regardless of the type of data. In relation to the balanced accuracy, a trend similar to that of the accuracy is observed, i.e., the CLR transformed data obtain higher values, are more robust and present less dispersion than the counts. Finally, with respect to the algorithms, it is observed that glmnet maintains high values for both accuracy and balanced accuracy in all the cohorts. Moreover, it is the algorithm with the lowest brier error in all the validation cohorts. This makes it a robust and generalisable algorithm.
